# Hierarchical mesoporous nickel cobaltite nanoneedle/carbon cloth arrays as superior flexible electrodes for supercapacitors

**DOI:** 10.1186/1556-276X-9-139

**Published:** 2014-03-24

**Authors:** Deyang Zhang, Hailong Yan, Yang Lu, Kangwen Qiu, Chunlei Wang, Chengchun Tang, Yihe Zhang, Chuanwei Cheng, Yongsong Luo

**Affiliations:** 1 School of Physics and Electronic Engineering, Xinyang Normal University, Xinyang 464000, People's Republic of China; 2 Key Laboratory of Advanced Micro/Nano Functional Materials, Xinyang Normal University, Xinyang 464000, People's Republic of China; 3 School of Material Science and Engineering, Hebei University of Technology, Tianjin 300130, People's Republic of China; 4 School of Materials Science and Technology, China University of Geosciences, Beijing 100083, People's Republic of China; 5 Shanghai Key Laboratory of Special Artificial Microstructure Materials and Technology, School of Physics Science and Engineering, Tongji University, Shanghai 200092, People's Republic of China; 6 Division of Physics and Applied Physics, School of Physical and Mathematical Sciences, Nanyang Technological University, Singapore 637371, Singapore

**Keywords:** Flexible, Supercapacitors, Nickel cobaltite, Nanoneedle, Carbon cloth

## Abstract

Hierarchical mesoporous NiCo_2_O_4_ nanoneedle arrays on carbon cloth have been fabricated by a simple hydrothermal approach combined with a post-annealing treatment. Such unique array nanoarchitectures exhibit remarkable electrochemical performance with high capacitance and desirable cycle life at high rates. When evaluated as an electrode material for supercapacitors, the NiCo_2_O_4_ nanoneedle arrays supported on carbon cloth was able to deliver high specific capacitance of 660 F g^-1^ at current densities of 2 A g^-1^ in 2 M KOH aqueous solution. In addition, the composite electrode shows excellent mechanical behavior and long-term cyclic stability (91.8% capacitance retention after 3,000 cycles). The fabrication method presented here is facile, cost-effective, and scalable, which may open a new pathway for real device applications.

## Background

Supercapacitors (SCs), also known as electrochemical capacitors, have attracted significant research attention due to their superior properties like high power density, excellent reversibility, and long cycle life for time-dependent power needs of modern electronics and power systems [1-9]. Especially, with the fast development of portable electronic devices with lightweight and flexible designs, the research on flexible storage devices becomes very important. The key research of supercapacitors is developing novel electrode materials with good specific capacitance and cycling stability plus high power density. It has been well established that nanostructured electrode designs can enhance both the power density (or rate capability) and cycling stability. Although a wide variety of nanostructures have been created and tested, it still represents a grand challenge to enhancing the capacity, maintaining the excellent rate capability and charge-discharge cycling life [10,11]. Ternary nickel cobaltite (NiCo_2_O_4_) has recently been investigated as a high performance electrode material for SCs because of its better electrical conductivity and higher electrochemical activity compared to binary nickel oxide (NiO) and cobalt oxide (Co_3_O_4_) [12]. Furthermore, NiCo_2_O_4_ with a broad range of morphologies were successfully fabricated, including three-dimensional (3D) urchin-like [13,14], monodisperse NiCo_2_O_4_ mesoporous microspheres [15], 2D nanofilms [16], mesoporous nanoflakes [17], nanosheets [18], 1D nanoneedle [19], nanowire [20-22], and porous nanotubes [23]. Therefore, NiCo_2_O_4_ has been conceived as a promising electrode material for SCs owing to its high specific capacitance, environmental compatibility, and cost-effectiveness.

In this communication, we demonstrate a rapid and facile method to prepare highly ordered 1D nanoneedle-like NiCo_2_O_4_ arrays on carbon cloth serving as electrode materials for SCs. Remarkably, the carbon cloth supported NiCo_2_O_4_ nanoneedles manifests ultrahigh SCs (660 F g^-1^ at 2 A g^-1^) and good cycling stability (91.8% capacitance retention after 3,000 cycles) at high rates in 2 M KOH aqueous electrolyte, making it a promising electrode for SCs. The fabrication method presented here is facile, cost-effective, and scalable, which may open a new pathway for real device applications [24,25].

## Methods

### Synthesis of NiCo_2_O_4_ nanoneedle arrays on carbon cloth

All the reagents were of analytical grade and directly used after purchase without further purification. Prior to deposition, commercial carbon cloths (1.5 × 4 cm in rectangular shape) were cleaned by sonication sequentially in acetone, 1 M HCl solution, deionized water, and ethanol for 15 min each, drying for standby. NiCo_2_O_4_ nanoneedle arrays (NCONAs) on carbon cloth were synthesized via a simple one-pot hydrothermal process. Four millimoles (1.1632 g) of Ni(NO_3_)_2_.6H_2_O and 8 mmol (2.3284 g) of Co(NO_3_)_2_.6H_2_O were dissolved into 75 mL of deionized water, followed by the addition of 15 mmol (0.9009 g) of urea at room temperature, and the mixture was stirred to form a clear pink solution. Then, the mixture was transferred in to a 100-mL Teflon-lined stainless autoclave. Then, the well-cleaned carbon cloth was immersed in the mixture, and the autoclave was kept at 120°C for 6 h. After it was cooled down to room temperature, the product supported on the carbon cloth was taken out and washed with deionized water and ethanol several times and cleaned by ultrasonication to remove the loosely attached products on the surface. After that, the sample was dried at 80°C for characterization. Finally, the as-prepared sample was annealed at 400°C in air for 2 h.

### Characterization

The crystalline structure and phase purity of the products were identified by X-ray diffraction (XRD) using a D8 Advance (Bruker, Karlsruhe, Germany) automated X-ray diffractometer system with Cu-Kα (*λ* = 1.5406 Å) radiation at 40 kV and 40 mA ranging from 10° to 70° at room temperature. Scanning electron microscopy (SEM) images were obtained using a Hitachi S-4800 microscope (Chiyoda-ku, Japan). Transmission electron microscopy (TEM) observations were carried out on a JEOL JEM-2010, Akishima-shi, Japan, instrument in bright field and on a high-resolution transmission electron microscopy (HRTEM) JEM-2010FEF instrument (operated at 200 kV). Raman spectra were carried out using WITec CRM200 Raman system, Ulm, Germany, equipped with a 532-nm laser source and a × 50 objective lens. The Brunauer-Emmett-Teller (BET) surface area of the NiCo_2_O_4_ nanoneedles was determined through nitrogen sorption measurement at 77K.

Electrochemical measurements were carried out by electrochemical workstation (CHI 660E, CH Instruments Inc., Shanghai, China) using three-electrode configuration in 2 M KOH aqueous solution. Both the pristine carbon cloth (≈1.5 × 4.0 cm^2^) and NCONAs (NiCo_2_O_4_ mass, ≈5 mg) were directly used as the working electrode. The value of specific capacitance (F g^-1^) and current rate (A g^-1^) was calculated based on the total mass of the active materials. The reference and counter electrodes were standard calomel electrode (SCE) and platinum foil, respectively. Cyclic voltammetry (CV) measurements were performed at a scanning rate of 2 to 40 mV s^-1^ from -0.2 to 0.6 V at room temperature. Galvanostatic charge-discharge measurements were carried out from -0.1 to 0.5 V at a current density of 2 to 16 A g^-1^, under opens circuit potential. Electrochemical impedance spectroscopy (EIS) measurements were performed by applying an alternate current (AC) voltage with 5 mV amplitude in a frequency range from 0.01 Hz to 100 kHz. The specific capacitances were calculated according to equation *C* = (*I*Δ*t*)/(Δ*V* × *m*), where *I* is the constant discharge current, Δ*t* is the discharge time, Δ*V* is the voltage drop upon discharging (excluding the IR drop), and *m* is the total mass of the active substance of the electrode material.

## Results and discussion

**Figure 1:**
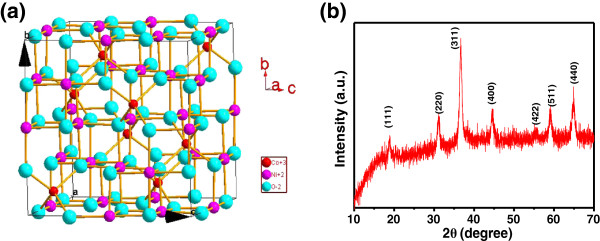
**Crystallographic structure and the crystallographic phase of NiCo**_**2**_**O**_**4 **_**with the spinel structure. (a)** Crystal structure of NiCo_2_O_4_. **(b)** XRD pattern of the NiCo_2_O_4_ nanoneedle arrays.

Figure 1 shows the crystallographic structure and the crystallographic phase of NiCo_2_O_4_ with the spinel structure. As depicted in Figure 1a, the Ni species occupy the octahedral sites and the Co is distributed over both octahedral and tetrahedral sites. Due to the presence of mixed valences of the same cation in such spinel cobaltite, the NiCo_2_O_4_ possesses at least 2 orders of magnitude higher electrical conductivity than that of the monometallic nickel and cobalt oxides by electron transfer taking place with relatively low activation energy between cations [12,26,27]. The crystallographic phase of the as-fabricated NCONAs product was studied by the XRD technique, and the typical wide-angle diffraction pattern is shown in Figure 1b (NCONAs were scraped from carbon cloth) and Additional file 1: Figure S2. Seven well-defined diffraction peaks, including not only the peak position but also their relative intensities, can be easily indexed as cubic spinel NiCo_2_O_4_ crystalline structure. In order to further understand the composition and structure of these NCONAs samples, Raman analysis was performed and the typical Raman spectrum of the products is shown in Additional file 1: Figure S1. In the Raman spectrum of carbon cloth, the G band (1,590 cm^-1^) represents the in-plane bond-stretching motion of the pairs of C sp^2^ atoms (the E_2g_ phonons), while the D band (1,350 cm^-1^) corresponds to breathing modes of rings or K-point phonons of A_1g_ symmetry [28]. Four peaks of the NCONAs at 187, 477, 523, and 671 cm^-1^ correspond to the F_2g_, E_g_, F_2g_, and A_1g_ models of NiCo_2_O_4_, respectively. These results are consistent with those documented in previous reports [29,30].

**Figure 2:**
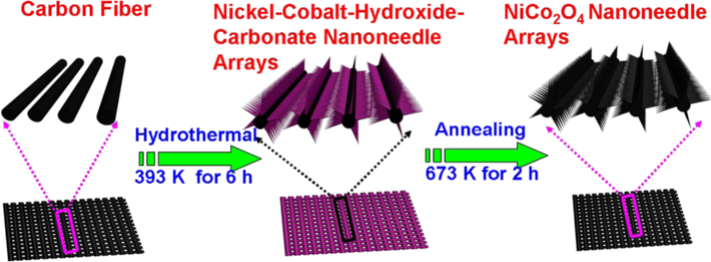
**Schematic illustration for the formation processes of the NiCo**_**2**_**O**_**4 **_**nanoneedles.**

**Figure 3:**
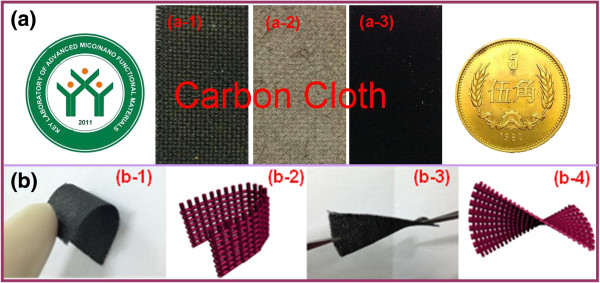
**The optical image of the flexible electrode material. (a)** The formation processes of the NCONAs growth on carbon cloth. **(b)** Optical images and schematic illustration for the flexible electrode material.

The schematic illustration of the fabrication process of NCONAs on carbon cloth substrate is shown in Figure 2. It can be seen that the whole process involves two steps: first, NCONAs precursor were longitudinally grown on the carbon cloth via a facile modified hydrothermal process according to previous work [19]; second, the obtained NCONAs precursor were subsequent post-annealing in air atmosphere; the color of the NCONAs precursor changed from dark gray to black, and the needle tip shape was still kept well. Moreover, Figure 3 is the optical image of the flexible electrode material. Figure 3a shows the optical image of the NCONAs in the formation processes. Meanwhile, carbon cloth can be readily rolled up as can be seen in Figure 3b, which is appropriate for flexible device applications.

**Figure 4:**
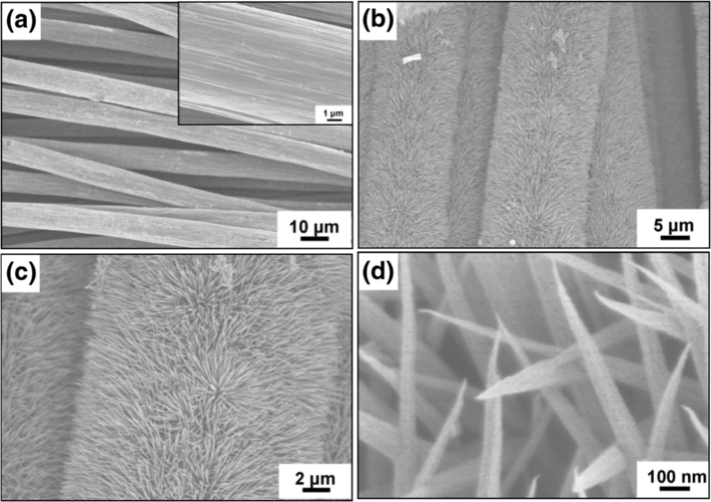
**Representative FESEM images of the well-cleaned carbon cloth and NCONAs grown on carbon cloth. (a)** High-magnification SEM images of the well-cleaned carbon fiber (the inset shows the surface of carbon fiber). **(b)** SEM image of carbon fiber after conformal coating of NCONAs. **(c,d)** High-magnification SEM image of NCONAs.

**Figure 5:**
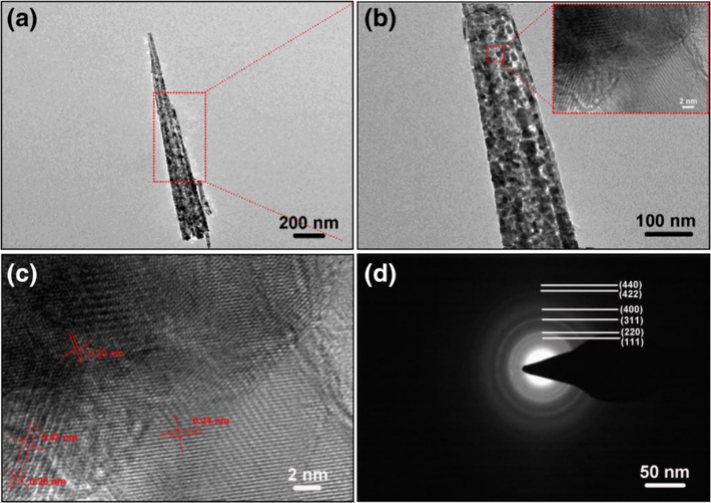
**TEM images and SAED patterns of the NCONAs. (a,b,c)** Low-magnification and high-magnification TEM images of the NCONAs. **(d)** The corresponding SAED patterns from NCONAs.

Figure 4a shows a SEM image of the well-cleaned carbon fibers, and the inset shows the details of the carbon fiber; we can see that the surface of the carbon fiber is smooth before the nanoneedle growth. After the nanoneedle growth, the surface of the whole carbon cloth becomes rough. Figure 4b,c,d demonstrates the higher magnification SEM images of NCONAs at different magnifications, indicating the growth of the target materials are large area and remarkably uniform, and provide clearer information about the carbon fiber growing NCONAs. From Figure 4b, it can be found that the as-obtained sample still reserved the 3D textile structure of the carbon fiber substrate, and the surface of each carbon fiber is uniformly covered with NCONAs. Further observation of an individual carbon fiber revealed that numerous NCONAs grew tidily and closely on the surface of the carbon fiber (Figure 4c,d). It is clear that the nanoneedle has a high aspect ratio, and from the high magnification SEM image in Figure 4d, we also can see that the NCONAs are of porous structures, which results from the release of gas during the decomposition of NCONAs precursor. Furthermore, the NCONAs have been ultrasonicated for several minutes before the FESEM examination, which confirms that the nanoneedles have a good adhesion on carbon cloth. The as-synthesized NCONAs are homogeneously aligned and separated apart adequately; it is expected that this unique structure might have a high surface area and high morphology stability and, consequently, could provide high specific capacitance due to the easy access of the active materials in the redox process to their interface, which are highly desirable for high-performance energy storage devices. More detailed information about the morphological and structural features of the as-synthesized NCONAs was studied by TEM, HRTEM, and selected area electron diffraction (SAED). From the dispersed nanoneedles as shown in Figure 5a,b, it can be seen that the nanoneedles possess sharp tips. The formation of the needle-like shape could be related to the depletion of precursor during the growth process. We also can see that the NCONAs are of porous structures in Figure 5b. HRTEM images reveal that nanocrystal domains are formed after thermal decomposition. A HRTEM image taken from a single nanocrystal within a nanoneedle is depicted in Figure 5c, confirming that the nanoneedles are of polycrystalline nature. The clearly resolved lattice fringes were calculated to be about 0.47, 0.28, 0.24, and 0.20 nm, corresponding to the (111), (220), (311), and (400) planes of spinel structured NiCo_2_O_4_. The SAED pattern depicted in Figure 5d further confirms the polycrystalline nature of the as-obtained NCONAs.

Electrode material with a large surface area is highly desirable for electrochemical SCs. The specific surface area and porous nature of the as-prepared nanoneedle-like NiCo_2_O_4_ nanostructures were further investigated by nitrogen adsorption-desorption measurements at 77 K. The nitrogen adsorption-desorption isotherm is an IV characteristic with a type H_2_ hysteresis loop in the range 0.8 to 1.0 p/p_o_ (Additional file 1: Figure S3), which might appear to be a unique characteristic of mesopores. The inset in the Additional file 1: Figure S3 shows the corresponding pore size distribution calculated by the Barrett-Joyner-Halenda (BJH) method from the desorption branch, indicating a narrow pore size distribution (10 to 30 nm) centered at around 12.4 nm. Thus, it can be concluded that the sample is characteristic of mesoporous materials. The specific surface area calculated by the BET method is *ca.* 44.8 m^2^ g^-1^ for the NCONAs.

**Figure 6:**
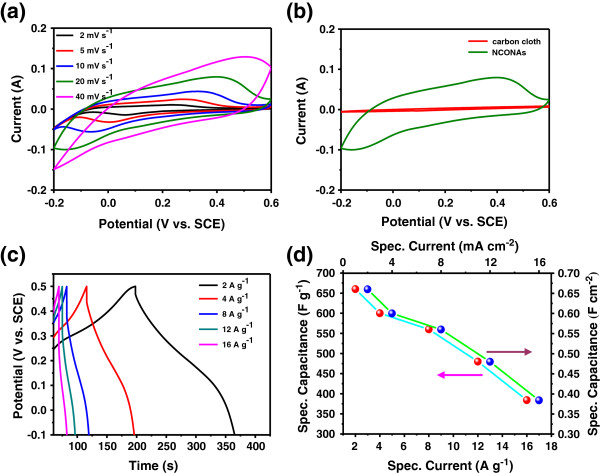
**Cyclic voltammograms, charge discharge curves, and specific capacitance of NCONAs. (a)** Cyclic voltammograms of NCONAs at different scan rates. **(b)** Cyclic voltammograms of the different electrode materials at 20 mV s^-1^. **(c)** Charge discharge curves of NCONAs at various current densities. **(d)** Current density dependence of the areal capacitance (right) and specific capacitance (left) of NCONAs.

**Figure 7:**
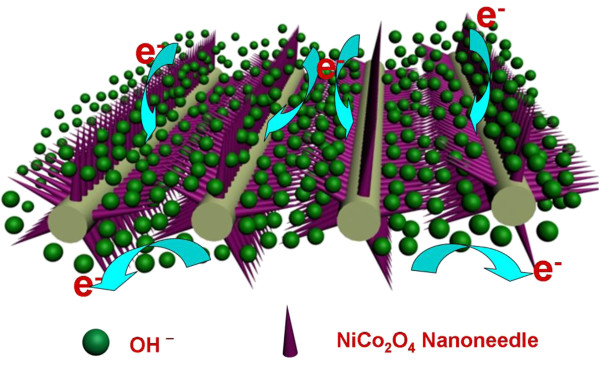
Schematic diagrams showing the kinetic advantages of the hybrid array in electrochemical energy storage.

As indicated by the BET results, these NCONAs with high specific surface area and porous structure may have potential applications in catalysis, sensors, and electrochemical SCs [31]. Herein, to characterize the electrochemical capacitive properties of the samples, the NCONAs as an integrated electrode were evaluated in three-electrode configuration with 2 M KOH aqueous solution as the electrolyte. Figure 6a shows the typical CV curves of the NCONAs electrode with various sweep rates ranging from 2 to 40 mV s^-1^. The shape of the CV curves clearly reveals the pseudocapacitive characteristics. Specifically, a pair of redox peaks can be observed within the potential range from -0.2 to 0.6 V (vs. SCE) for all sweep rates, which is mainly related to the faradaic redox reactions related to M-O/M-O-OH (M = Co and Ni ions) in the alkaline electrolyte (Figure 7), as shown in the following equations [32-34]:

(1) $${\mathrm{NiCo}}_{2}O_{4}+{\mathrm{OH}}^{‐}+H_{2}O↔\mathrm{NiOOH}+2\mathrm{CoOOH}+e^{‐},$$

(2) $$\mathrm{CoOOH}+{\mathrm{OH}}^{‐}↔{\mathrm{CoO}}_{2}+H_{2}O+e^{‐},$$

The peaks are located at around 0.05 and 0.25 V (vs. SCE) when the scan rate is 2 mV s^-1^. With the 20-fold increase in the sweep rate from 2 to 40 mV s^-1^, the position of the cathodic peak shifts from 0.05 to -0.15 V (vs. SCE). This indicates the low resistance of the electrode because of the conductive carbon cloth substrate [19]. For comparison, the CV of the pristine carbon cloth and NCONAs electrode at 20 mV s^-1^ are also shown in Figure 6b. It is noted that the area of the curve of the NCONAs electrode at the same scan rate is higher than that of the carbon cloth electrode materials. The significant increase of the CV integrated area suggests that the nanoneedle-like NiCo_2_O_4_ arrays have a much higher specific capacitance, as will be discussed. Therefore, the excellent electrochemical capability of NCONAs may be attributed to their unique microstructures. From the constant current discharge profiles (Figure 6c), it can be observed that there are voltage plateaus at around 0.2 to 0.15 V (vs. SCE), which is consistent with previous literature [22,35]. Specific and areal capacitances were calculated using Equations 3 and 4, respectively.

(3) $$C_{\mathrm{sp}}=I\times \Delta t/\left(m\times \Delta V\right),$$

(4) $$C_{a}=I\times \Delta t/\left(S\times \Delta V\right),$$

where *I* (mA) represents the constant discharge current, *m* (mg), Δ*V* (V), and Δ*t* (s) designate the mass of active materials, potential drop during discharge (excluding the IR drop), and total discharge time, respectively. *S* is the nominal area of CC covered with NCONAs (about 5 cm^2^). The calculated areal capacitance as a function of the discharge current density is plotted in Figure 6d. On the basis of the above results, the specific capacitance of the NCONAs at 2, 4, 8, 12, and 16 A g^-1^ is 660, 600, 560, 480, and 384 F g^-1^, respectively. About 58.2% of specific capacity was retained when the current density increased from 2 to 16 A g^-1^. It is emphasized that the specific capacitance is calculated according to the mass of the NCONAs, and carbon materials are not included for this calculation. The areal capacitance is as high as 0.660, 0.600, 0.560, 0.480, and 0.384 F cm^-2^ measured at the discharge current density of 2, 4, 8, 12, and 16 mA cm^-2^, respectively.

**Figure 8:**
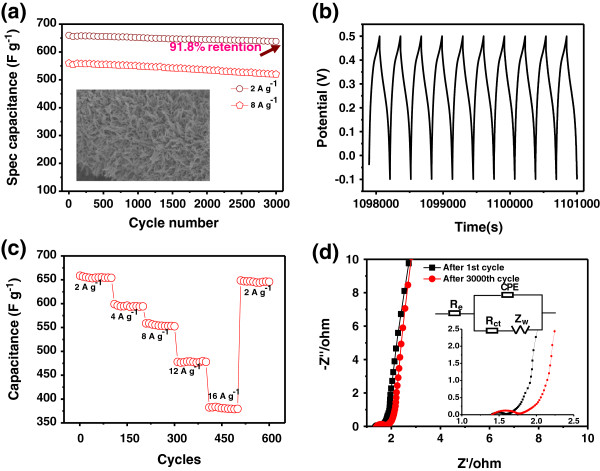
**Cycling performance and electrochemical impedance spectra of the NCONAs supercapacitor. (a)** Cycling performance of the NCONAs supercapacitor device over 3,000 cycles at 2 and 8 A g^-1^ (inset, the SEM of the NCONAs after 3,000 cycles at 2 A g^-1^). **(b)** The charge/discharge curves of the last 10 cycles during in 3,000 cycles for the NCONAs. **(c)** Cycling stability of the NCONAs at progressively various current densities. **(d)** Electrochemical impedance spectra after 1st and 3,000th cycles of NCONAs.

The cycle stability of SCs is a crucial parameter for their practical applications. The long-term stability of the electrodes was examined at 2 and 8 A g^-1^, and the results are shown in Figure 8a. It is found that the NCONAs electrodes capacitance retention is about 91.8% of initial value after 3,000 cycles at 2 A g^-1^. As illustrated in the inset of Figure 8a, the NCONAs structures were well maintained and overall preserved with little structural deformation after 3,000 cycles. The NCONAs electrode exhibits a good long-term electrochemical stability which is further evident from the very stable charge/discharge curves for the last 10 cycles (Figure 8b). The results indicated that the charge curves are still very symmetric to their corresponding discharge counterparts, showing no significant structural change of the NCONAs electrode during the charge/discharge processes.

Furthermore, for a better understanding of the synergistic effect in this electrode design, the cycling performance of the NCONAs at progressively increased current density was recorded in Figure 8c. During the first 100 cycles with a charge discharge density of 2 A g^-1^, the hybrid structure shows a cycle stability performance and the specific capacity as high as 658 F g^-1^. In the following cycles, the charge/discharge rate changes successively; the hybrid structure always demonstrates stable capacitance even suffering from sudden change of the current delivery. With the current rate back to 2 A g^-1^ for the rest of cycles, a capacitance of approximately 656 F g^-1^ can be recovered and without noticeable decrease, which demonstrates the hybrid structure has excellent rate performance and cyclability. The loss of specific capacitance may result from ineffective contacts between part of the unstable NCONAs and the following deterioration of the electron transfer and ion diffusion.

To further show the merits of the NCONAs and CC composite material as the electrode material, EIS provided beneficial tools to reveal the electronic conductivity during the redox process. Impedance spectra of the NCONAs electrode material were measured at open circuit potential with an AC perturbation of 5 mV in the frequency range from 0.1 Hz to 10^3^ KHz. Nyquist plots in Figure 8d were composed of an arc in the high-frequency region and a nearly straight line in the low-frequency region. Herein, the high-frequency intercept with the *X*-axis represented the equivalent series resistance (*R*_s_), associated with the sum of the electrolyte solution resistance, the intrinsic resistance of active material, and the contact resistance at the electrode-electrolyte interface. The charge transfer resistance of electrode (Rct) was calculated from the diameter of the semicircle in the high-frequency region, while the straight line at lower frequencies presented the diffusion behavior of ions in the electrode pores. The steeper shape of the sloped line represented an ideal capacitive behavior with the faster diffusion of ions in electrolyte [36]. The measured impedance spectra were analyzed using the complex nonlinear least-squares fitting method on the basis of the equivalent circuit, which is given in the inset of Figure 8d. From the magnified high-frequency regions in the inset of Figure 8d, the NCONAs electrodes after 1st and 3,000th cycles show the charge transfer resistances (*R*_ct_), respectively. The *R*_ct_ value increases only slightly from 1st and 3,000th cycles owing to good contact between the current collector and nanoneedle arrays. These analyses revealed that the good electrical conductivity and ion diffusion behavior resulted in the high performance of NCONAs carbon cloth composite as electrode material for SCs.

Based on abundant electrochemical analysis, owing to the synergistic effects between nanoneedle arrays and carbon cloth, the flexible NCONAs and carbon cloth composite electrode material exhibit high specific capacitance. The improved electrochemical performance could be related to the following structural features. Firstly, large surface areas facilitate ion diffusion from the electrolyte to each NCONA, making full use of the active materials, which undoubtedly contributes to the high capacitance. Secondly, carbon cloth in the hybrid materials could provide not only double layer capacitance to the overall energy storage but also fast electronic transfer channels to improve the electrochemical performances [29]. Third, the direct growth of NCONAs on a conductive substrate could ensure good mechanical adhesion, and more importantly, good electrical connection with the conductive substrate that also serves as the current collector in such binder-free electrodes [35,37]. In this way, the decreased ion diffusion and charge transfer resistances lead to the improved specific capacitance. Meanwhile, the synergistic effects result in the better cycling stability of the NCONAs and carbon cloth composite electrode. NCONAs in a vertical array and carbon cloth as the platform for sustaining nanoneedles arrays withstand the strain relaxation and mechanical deformation, preventing the electrode materials from seriously swelling and shrinking during the insertion-deinsertion process of the counter ions [38,39].

## Conclusions

In summary, we have presented a facile and high-efficiency hydrothermal method for the direct growth of NCONAs on flexible substrates. The synthesis route presented here is robust and may be extended to fabricate other nanostructures for various applications in electrochemical energy storage and optical devices. The NCONAs supported on carbon cloth were tested as highly flexible SCs, and they have demonstrated excellent electrochemical performance; also, they have superior cycling stability that can maintain good performance over 3,000 cycles. Our as-fabricated SCs electrode material demonstrate their feasibility as efficient energy storage devices. Our work here opens up opportunities for flexible energy storage devices in future wearable devices area and many other flexible, lightweight, and high-performance functional nanoscale devices.

## Competing interests

The authors declare that they have no competing interests.

## Authors' contributions

DZ carried out the sample preparation, performed all the analyses, and wrote the paper. YL (Lu), and KQ participated on its analysis. HY, CW, CC, CT, YZ, and YL (Luo) directed the research and made corrections to the manuscript. All authors read and approved the final manuscript.

## Supplementary Material

Additional file 1 **Supporting information. Figure S1.** Raman spectra of NCONAs. **Figure S2.** XRD patterns of NiCo_2_O_4_ nanoneedles/carbon cloth composite. **Figure S3.** Nitrogen adsorption-desorption isotherm and the corresponding pore size distribution of mesoporous NCONAs. Click here for file
