# Supplementary material for: Hierarchical mesoporous nickel cobaltite nanoneedle/carbon cloth arrays as superior flexible electrodes for supercapacitors
Source: Nanoscale Res Lett. 2014 Mar 24;9(1):139. doi: 10.1186/1556-276X-9-139 (PMC3987927; doi:10.1186/1556-276X-9-139)
Supplement: Additional file 1 — Supporting information. Figure S1. Raman spectra of NCONAs. Figure S2. XRD patterns of NiCo2O4 nanoneedles/carbon cloth composite. Figure S3. Nitrogen adsorption-desorption isotherm and the corresponding pore size distribution of mesoporous NCONAs. [file 1556-276X-9-139-S1.doc]

**Supporting Information**

**Hierarchical Mesoporous Nickel Cobaltite Nanoneedle/ Carbon cloth Arrays as Superior Flexible Electrodes for Supercapacitors**

Deyang Zhang^†^*^1,2^*

Email: zhangdeyang329@163.com

Hailong Yan^†^*^1,2^*

Email: ehlyan@163.com

Yang Lu^†^*^1,2,3^*

Email: luyang.181@163.com

Kangwen Qiu^†^*^1,2^*

Email: ekwqiu@163.com

Chunlei Wang*^1,2^*

Email: [eclwang@163.com](mailto:eclwang@163.com)

Chengchun Tang*^3^*

Email: [tangcc@hebut.edu.cn](mailto:tangcc@hebut.edu.cn)

Yihe Zhang*^4^*

Email: [zyh@cugb.edu.cn](mailto:zyh@cugb.edu.cn)

Chuanwei Cheng*^5^*

Email: [cwcheng@tongji.edu.cn](mailto:cwcheng@tongji.edu.cn)

Yongsong Luo*^1,2,6*^*

* Corresponding author

Email: [ysluo@xynu.edu.cn](mailto:ysluo@xynu.edu.cn)

^†^These authors contribute equally to this work

*^1^ School of Physics and Electronic Engineering, Xinyang Normal University, Xinyang 464000, P. R. China*

*^2^ Key Laboratory of Advanced Micro/Nano Functional Materials, Xinyang Normal University, Xinyang 464000, P. R. China*

*^3^ School of Material Science and Engineering, Hebei University of Technology, Tianjin 300130, P. R. China*

*^4^ School of Materials Science and Technology, China University of Geosciences, Beijing 100083, P. R. China*

*^5^ Shanghai Key Laboratory of Special Artificial Microstructure Materials And Technology, School of Physics Science and Engineering, Tongji University, Shanghai 200092, P.R. China*

*^6^ Division of Physics and Applied Physics, School of Physical and Mathematical Sciences, Nanyang Technological University, 637371, Singapore*

**Figure S1** Raman spectra of NCONAs. The spectrum of pure carbon cloth is also shown for comparison.

**Figure S2** XRD pattern of NiCo_2_O_4_ nanoneedles/carbon cloth composite.

**Figure S3** Nitrogen adsorption−desorption isotherm and the corresponding pore size distribution (inset) of mesoporous NCONAs.
